# Treatment outcome of supervised exercise, home exercise and bite splint therapy, respectively, in patients with symptomatic disc displacement with reduction: A randomised clinical trial

**DOI:** 10.1111/joor.12888

**Published:** 2019-09-30

**Authors:** Anders Wänman, Susanna Marklund

**Affiliations:** ^1^ Department of Odontology Clinical Oral Physiology Umeå University Umeå Sweden; ^2^ Department of Clinical Oral Physiology Västerbotten County Council Umeå Sweden

**Keywords:** home care, motor activity, oral appliance, temporomandibular disorders, temporomandibular joint dysfunction

## Abstract

The best treatment strategy for disturbing temporomandibular clicking sounds is not known. The aim was to evaluate the effect of exercise and bite splint therapy, respectively, in patients with symptomatic disc displacement with reduction. The study was a randomised clinical trial of subjects with temporomandibular joint (TMJ) clicking sounds with a reported severity/intensity of ≥4 on a numerical rating scale (0‐10) and signs fulfilling the Research Diagnostic Criteria (RDC/TMD) for disc displacement with reduction. Thirty subjects each were randomised to bite splint, home exercise, or supervised exercise programme at the clinic. Two examiners (authors), blinded to the treatment modality, examined the same subject at baseline and at a 3‐month follow‐up. Non‐parametric statistical methods were applied for analyses. A *P*‐value <.05 was considered statistically significant. The dropout rate was highest in the home exercise group. About 50% of the participants reported improvement of their TMJ sounds with no significant difference between treatments. In the supervised exercise and the bite splint groups, approximately 2/3 of the patients reported 30% or more improvement of their TMJ sounds and half reported 50% improvement or more. The supervised exercise group also showed reductions in TMD pain, neck disability, mood disturbances and somatisation. Jaw exercise programmes and bite splint treatments had positive effects on TMJ clicking. The supervised exercise programme had an additional effect on the subject's well‐being and thus may help to encourage patient's empowerment and coping strategies.

## 1 BACKGROUND

Temporomandibular disorders (TMD) involve symptoms such as pain around the temporomandibular joint (TMJ) and jaw muscles, pain on jaw movements, impaired jaw mobility, TMJ sounds, and temporary or persistent locking of the jaw.^1^ The symptoms affect individual's wellbeing and quality of life.^2^, ^3^ Most intervention studies in this field have been directed towards pain conditions and only a few^4^, ^5^ have focused on the most commonly reported TMD condition in the population, that is symptomatic disc displacement with reduction. This condition commonly involves clicking sounds caused by interference of the TMJ disc, and treatment may in some specific cases end up in discectomy. TMJ clicking sounds with or without associated pain can thus significantly disturb the individual's normal jaw function and generate a demand for treatment. Reversible treatment modalities are recommended as primary intervention for patients with TMD. These modalities include advice, bite splints, jaw exercises and over‐the‐counter medication. The National Guidelines for dental health care in Sweden^6^ clearly revealed that robust science based on randomised clinical trials (RCT) is generally lacking, and consequently, this affects the clinical decision‐making. Even though published studies indicate some beneficial effects, these studies are heterogeneous in composition of study populations, diagnoses, treatment modalities and outcome measures. Other drawbacks are lack of description of intention‐to‐treat measures, reasons for dropouts, evaluation of patient's compliance, use of rescue medication, side‐effects, etc. Thus, there are currently uncertainties about the effects of different treatment modalities, and well‐designed RCTs are needed to improve the scientific base of treatment outcome in patients hampered by TMD.

The overall objective was to abate the current uncertainties about the effects of treatment modalities for symptomatic disc displacement with reduction. The primary aim was to evaluate the effect of supervised and home exercise programmes, respectively, among patients with symptomatic disc displacement with reduction. The second aim was to evaluate whether there is a significant difference in outcomes between a supervised exercise programme, home exercise programme and bite splint therapy. Bite splint therapy was used as “standard treatment.” Our first hypothesis was that a supervised exercise programme has a moderate to high effect on symptoms frequency and intensity, as well as on jaw opening capacity. Further, this effect was expected to be significantly better than a home exercise programme. The second hypothesis was that treatment with a bite splint has a moderate effect on TMJ symptoms and does not differ significantly from a supervised exercise programme; also, a low effect on jaw opening capacity was expected. The third hypothesis was that a home exercise programme has low‐to‐moderate effect on symptom relief that is significantly lower than bite splint and a supervised exercise programme.

## 2 METHODS

### 2.1 Study design

The study design was a randomised controlled clinical trial (RCT) with three parallel treatment groups of 30 subjects in each group. The participants were examined at baseline and re‐examined after 3 months. The study was approved by the Regional Ethical Review Board in Umeå, Sweden, (Dnr 2011‐219‐31M) and carried out in accordance with the Declaration of Helsinki.

### 2.2 Study population

Participants were recruited from patients referred to the Clinical Oral Physiology department in Umeå, Sweden as well as among those who responded to targeted advertising. After a screening procedure, all subjects fulfilling the inclusion criteria were given oral and written information about the study. Those who were willing to participate signed an informed consent. In total, 593 individuals were screened and 90 subjects were included in the trial.

The general inclusion criteria were age between 18 and 70 years, accommodation in Umeå Municipality's proximity, and able to understand Swedish, orally and written. They should have no major psychiatric diagnosis, no ongoing dental, medical or physiotherapeutic treatments related to the patient's symptom that may interfere with the study, no active rheumatologic disease and no malignant disease.

The specific inclusion criteria for being allocated to symptomatic disc displacement with reduction were that TMJ clicking sounds were presented as their major symptom. They should fulfil the Research Diagnostic Criteria for TemporoMandibular Disorders (RDC/TMD) for symptomatic disc displacement (ie, reproducible TMJ clicking sounds during jaw opening and closing with the opening click registered at >5 mm interincisal distance and with TMJ clicking sounds that cannot be recognised when jaw opening is performed in a protruded position). The participants should indicate the severity of the TMJ clicking sounds at ≥4 on a Numerical Rating Scale (NRS 0‐10) and the frequency of TMJ clicking sounds once a week or more.

### 2.3 Study settings

The study was performed at the Department of Odontology/Clinical Oral Physiology at Umeå University Sweden in collaboration with the Public Dental Health service. Two specialists in clinical oral physiology/TMD performed the examinations. Each participant had the same examiner at baseline and at 3 months follow‐up. The examiner was always blinded to the participant's intervention. Two assistants were engaged in the treatments.

### 2.4 Questionnaire

After enrolment, the participants received the Swedish translation of the RDC/TMD questionnaire, supplemented with the following questions: Jaw function limitation scale‐20 (JFLS‐20), Neck Disability Index (NDI) and rating of the subject's motivation to complete the intervention on NRS 0‐10.

The questionnaire was filled out at the subject's home and was taken to the clinic where subjects were randomised to intervention.

### 2.5 Randomising process and blinding

Those who fulfilled the inclusion criteria were randomised into three different treatment groups. There were 30 individuals in each group. The randomisation was done with the aid of SPSS 20 (randomised numbers) before the study started. When a subject fulfilled the inclusion criteria and accepted to participate, an assistant contacted the subject and assigned the participant to intervention. Participants received written and oral information about the study and signed an informed consent. Each participant was carefully instructed to not disclose his/her treatment allocation to the examiner at follow‐up. All treatments were performed by trained assistants that were not involved in the examination and evaluation of the respective participant. The two examiners (authors) were blinded to the subject's intervention trial. They examined the same subject clinically at follow‐up the same as at baseline.

### 2.6 Interventions

All patients received brief oral information of the TMJ structure and function that was performed by the same assistant. Subjects were instructed to avoid food with a tough texture and to try to not provoke TMJ clicking sounds during the day.

- Bite splint. One group (20 women and 10 men, mean age 40.4 years, SD 17.0) received a resilient bite splint, 4 mm thick BIOPLAST^®^ (Scheu Dental GmbH) produced in a BIOSTAR^®^ heat and vacuum press (Scheu Dental GmbH). The bite splint was adjusted by a trained assistant not involved in the evaluations of the participants to have a flat surface with occlusal contacts in molar, premolar and canine regions. The appliance should be used during sleeping. The subject was contacted by phone after 1 week to check whether the appliance was accepted and used. After 6 weeks, the assistant checked the function of the appliance and readjusted it if needed. The patient was encouraged to continue to use the appliance.
- Home exercise. One group (24 women and six men, mean age 38.5 years, SD 14.4) received a home regime of jaw exercises. They were instructed to do two different trainings. The 1st type of exercise was jaw opening and closing movements daily for 5 minutes after each meal with the mandible and the head of the TMJ in a slightly protruded position according to Yoda et al.^5^ This exercise should not produce any clicking sounds. The 2nd type of exercise was isometric exercises according to Au and Klineberg^4^; this entailed jaw opening and jaw protrusion against resistance with the hand for 10 seconds with 10 repetitions each daily. Participants received a training brochure with photos of the exercises and detailed information on frequency and exercise time. The brochure also included a diary. After 6 weeks, the subject and the assistant checked the performance of the exercise and the diary. The patient was encouraged to continue to do the exercises on a daily basis.
- Supervised exercise. One group (19 women and 11 men, mean age 37.1 years, SD 14.1) received a supervised exercise programme that included 10 sessions. The 1st exercise station was a 5‐minute warming up of the jaw with a heat lamp. The 2nd station involved jaw opening‐closing movements (TMJ rotation) with the mandible in a slightly protruded position for 6 minutes. The 3rd and 4th exercise stations comprised jaw opening and jaw protrusion, respectively, against resistance for 4 minutes each. After 10 sessions, subjects received the same brochure as in group B and were encouraged to continue the exercises at home.

After 3 months of treatment, all patients received a new, blinded clinical examination and evaluation by the same examiner as at baseline.

### 2.7 Outcomes

The outcomes were based on the domains recommended by IMMPACT^7^ and the CONSORT statement: symptoms, physical functioning, emotional functioning, participant's ratings of global improvement, adverse events and participant's disposition. Measurements were done before the trial started and after 3 months. The outcomes were based on response to the questionnaire and observations at the clinical examinations. The frequency of TMJ clicking sounds, TMJ locking and jaw pain, was stated, respectively, on a 5‐graded scale (never = 0, occasionally = 1, once or twice a month = 2, once a week = 3, several times a week = 4, and daily = 5). Each symptom's intensity was stated on NRS (0‐10) that was anchored as no problem (0) in one end and maximal/unbearable (10) in the other end. A symptom index was calculated as the product of symptom frequency and intensity (0‐50). A reduction of the symptom index of 30% or more was considered moderate improvement and a reduction of 50% or more as substantial improvement. Jaw function limitation scale‐20, signs of depression, signs of somatisation, Neck Disability Index (NDI) and the Patient Global Impression of Change (PGIC). Changes in the maximal jaw movement capacity in mm were also included as outcome measures.

### 2.8 Sample size

A power calculation based on the results by Burgess et al^8^ revealed that 21 subjects in each subgroup would be sufficient to detect a difference when *α* = 0.05 and *β* = 0.8. With an expected dropout of 25%, the number for each trial was decided at 30 subjects. Thus, a total of 90 subjects were registered for the trials.

### 2.9 Statistical methods

Analyses were based on an intention‐to‐treat approach. The individual baseline data were imputed for dropouts regardless of reason. Changes were calculated as the difference between baseline data and data at the 3‐month follow‐up. Variables with normal distribution were analysed with independent sample *t* test, and the remaining with non‐parametrical Wilcoxon for paired observations and Chi‐square. A *P*‐value <.05 was considered statistically significant.

## 3 RESULTS

**Figure 1:**
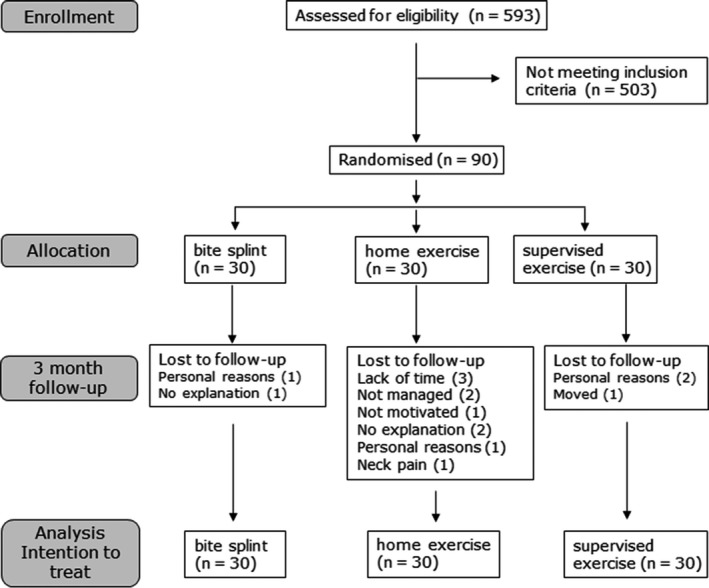
Participant's flow, loss to follow up and reasons for dropping out. In the analysis was the baseline data imputed at follow‐up for dropouts

Flow chart of number of participants, loss to follow‐up and reasons for dropout are presented in Figure 1. The number of dropouts was highest among the home exercise group (n = 10), compared with the bite splint group (n = 2) and supervised exercise group (n = 3). There were more women (n = 63) than men (n = 27) included in the study, with no significant difference between treatment groups. The mean age of the participants was 39.2 years (SD 15.2) with no significant difference between the groups. There was no significant difference between the treatment groups regarding stated motivation for treatment (mean 8.7, SD 1.6) either among dropouts or among those who fulfilled the treatment period until the 3‐month follow‐up.

**Figure 2:**
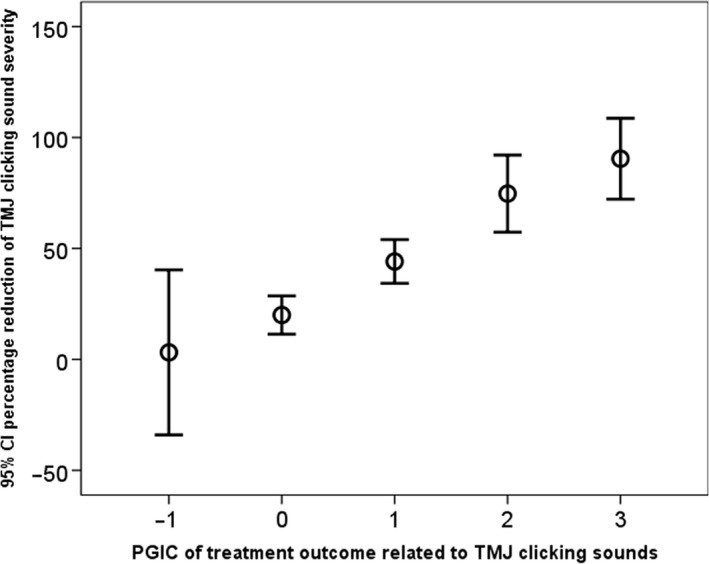
95% confidence interval (CI) of the percentage reduction of temporomandibular joint (TMJ) severity in relation to patient's global impression of change (PGIC). Worse = −1, no change = 0, minimally improved = 1, much improved = 2, very much improved = 3

Before treatment, the reported mean severity of TMJ sounds ranged 33.4‐34.0 on the 50‐graded scale in the three treatment groups. The severity level was significantly reduced in all three treatment groups (post vs pre). The reduction was significantly larger in the bite splint and supervised exercise groups compared with home exercise (Table 1). Registered TMJ clicking disappeared in between 17% and 30% of the cases, and the reduction was significant in all treatment groups (Table 2). Almost half of the participants reported improvement of their TMJ sounds (PGIC) with no significant difference between the treatment modality. A few participants reported impairment of their TMJ sounds or a transition to TMJ locking (Table 3). In the bite splint group and in the supervised exercise group, approximately 2/3 reported 30% or more improvement of their TMJ sounds and half reported 50% improvement or more (Table 3). The relationship between PGIC for TMJ clicking sound and the percentage reduction of TMJ clicking severity is presented in Figure 2. The relationship was significant (*P* < .001; ANOVA).

**Table 1:** Distribution of pre‐ and post‐treatment values for frequent temporomandibular disorders symptoms, severity of temporomandibular joint (TMJ) symptoms, neck disability index (NDI) and Jaw Function Limitation Scale 20 (JFLS‐20) associated with the following treatment modalities in a blinded randomised control trial: bite splint, home exercise and supervised exercise

Symptoms and symptom indices	Bite splint (BS)		Home exercise (HE)		Supervised exercise (SE)		Δ Pre‐post
	Pre/post (n = 30)	*P*‐value^a^	Pre/post (n = 30)	*P*‐value^a^	Pre/post (n = 30)	*P*‐value^a^	BS:HE:SE *P*‐value^b^
TMJ clicking sounds (%)	100/100		100/93		100/93		
Locking of the jaw (%)	62/35	.02	44/37		55/38		
Pain in jaw, TMJ, temples (%)	50/30		58/50		63/38	.008	
Pain in jaw, TMJ, temples during jaw movements (%)	50/33		44/43		69/33	.002	HE vs SE .003
Severity of TMJ sounds (0‐50) mean (SD)	33.4 (11.8)/18.8 (9.7)	<.001	33.8(11.5)/26.7(16.4)	.004	34.0 (9.7)/18.8 (10.6)	<.001	BS vs HE .02 HE vs SE .02
Severity of TMJ locking (0‐50) mean (SD)	13.3 (16.4)/6.2 (11.8)	.003	9.4(13.4)/7.1(11.1)		10.6 (13.7)/5.6(10.2)	.023	
Severity of jaw pain (0‐50) mean (SD)	7.8 (10.0)/3.5 (7.2)	.03	8.3 (9.1)/7.2 (9.7)		12.0 (9.9)/7.1 (10)	.007	
NDI mean (SD)	8.1 (7.6)/6.8 (5.9)		11.4(10.7)/10.3(12.4)		10.0 (10.8)/7.9 (7.8)	.016	
Depression sum mean (SD)	0.46 (0.3)/0.38 (0.3)		0.66 (0.6)/0.63(0.7)		0.54 (0.5)/0.34(0.3)	.001	HE vs SE .04
Somatisation sum mean (SD)	0.45 (0.4)/0.45 (0.3)		0.63(0.5)/0.48(0.5)	.02	0.52 (0.5)/0.36 (0.3)	.003	
JFLS‐20 mean (SD)	16.3 (18.2)/16.0(13.1)		18.9(33.3)/14.6(16.4)		18.5 (15.5)/14.0 (13.0)		

Note

Pre = baseline data/post = 3‐mo treatment follow‐up data.

a Wilcoxon's test for paired observations.

b Independent sample *t* test.

**Table 2:** Pre‐ and post‐treatment values for registered temporomandibular disorders clicking sounds, and maximal jaw mobility, associated with the following treatment modalities in a blinded randomised control trial: bite splint, home exercise and supervised exercise

Signs	Bite splint (BS)		Home exercise (HE)		Supervised exercise (SE)		Δ Pre‐post
	Pre/post (n = 30)	*P*‐value^a^	Pre/post (n = 30)	*P*‐value^a^	Pre/post (n = 30)	*P*‐value^a^	BS:HE:SE *P*‐value^b^
TMJ clicking sounds (%)	100/83	.03	100/80	.02	100/70	.003	
Jaw opening (mm) mean (SD)	46.2 (6.3)/46.8 (6.0)		48.9 (5.0)/48.1 (5.5)		49.9 (6.6)/49.5 (7.5)		
Laterotrusion right (mm) mean (SD)	10.2 (2.0)/10.5 (1.9)		10.1 (2.0)/10.1 (2.1)		10.0 (2.1)/10.2 (1.7)		
Laterotrusion left (mm) mean (SD)	9.0 (2.7)/9.9 (1.7)	.01	9.3 (2.4)/9.3 (1.7)		9.1 (1.9)/9.5 (1.8)		
Protrusion (mm) mean (SD)	9.0 (2.1)/8.7 (1.9)		9.0 (2.4)/9.1 (2.4)		9.1 (2.2)/9.4 (1.9)		

Note

Pre = baseline data/post = 3‐mo treatment follow‐up data.

a Wilcoxon's test for paired observations.

b Independent sample *t* test.

**Table 3:** Percentage distribution of Patients Global Impression of Change (PGIC) of temporomandibular joint (TMJ) clicking sounds and TMJ locking associated with the following treatment modalities in a blinded randomised control trial: bite splint, home exercise and supervised exercise

	PGIC	Bite splint (n = 30)		Home exercise (n = 30)		Supervised exercise (n = 30)		*P*‐value^a^
		(n)	(%)	(n)	(%)	(n)	(%)	
TMJ clicking sounds	Impaired	1	3	3		1	3	.6
	Unchanged	16	53	14	47	12	40	
	Improved	13	44	13	43	17	57	
Locking of the jaw	Impaired	1	4	0	0	1	3	.6
	Unchanged	18	60	23	77	18	60	
	Improved	11	37	7	23	11	37	

	Improved symptom severity	(n)	(%)	(n)	(%)	(n)	(%)	
TMJ clicking sounds	≥30% improved	21	70	9	30	19	63	.004
	≥50% improved	15	50	8	27	15	50	.1

a Kruskal‐Wallis test.

Temporomandibular disorders pain was a co‐morbid symptom for 60% of these cases with TMJ clicking sounds as predominate disorder and 46% reported at the start of the study awareness of tooth clenching or tooth grinding at least once a week or more. No neck disability (NDI <10%) was reported by 63%, and no limitation of jaw function (JFLS <5) was reported by 1/3 of the total sample. After the 3‐month intervention period showed the supervised exercise group the highest individual improvements for the concomitant symptoms; TMD pain, neck disability, mood disturbances and somatisation (Table 1).

## 4 DISCUSSION

In this study, we specifically addressed the condition symptomatic disc displacement with reduction, with specific focus on the question of whether treatment with exercise programmes or a bite splint could reduce disturbing TMJ clicking sounds. A few studies have specifically addressed annoying clicking sounds even if these can pose a significant handicap for the individual with or without associated pain. For some patients, surgery is the treatment of demand. In our study, the first hypothesis was met, that is, that a supervised exercise programme showed a moderate to high effect of reducing the reported severity of TMJ clicking at a 3‐month follow‐up. The exercise programme had no effect, either positive or negative, on jaw opening capacity. A moderate improvement (≥30%) was more often observed after the supervised exercise programme compared with the home exercise programme. The second hypothesis that the use of a bite splint would have a similar effect on TMJ clicking sounds as the supervised exercise programme was largely confirmed. The third hypothesis that a home exercise programme would show a lower effect on symptom relief compared with the other two groups was partly rejected; the effect was low but subjects in this group also reported reduced severity of the TMJ clicking sounds, and for 1/3, the baseline values had to be imputed due to dropout. A behaviour change depends on the individuals' willingness, empowerment, motivation and self‐efficacy. The participants' statement of motivation at the start of the study was high, and so was their willingness to conduct the study.

Our results can be interpreted in many ways. The ability of the treatment regimens to eliminate TMJ clicking was low since almost all patients reported persistent TMJ clicking at the follow‐up. On the other hand, the reported perceived severity of the TMJ sounds was reduced in all three treatments groups. This reduction indicates that the sounds, in the patient's own opinion, occurred less frequently or at a lower intensity or both. The strong relationship between the patient's global impression of change (PGIC) and the percentage change of the severity level supports the finding. These results are similar to outcomes of chronic pain conditions reported by Farrar et al 2001.^9^ These authors concluded that a reduction of approximately 30% of pain intensity represents a clinically important difference. It is not known if that level can be transposed to non‐painful symptoms such as disturbing TMJ clicking. An interpretation of the results from this study may be that a 50% reduction or more of the severity index may indicate important improvement for patients with TMJ sounds. A qualitative study design may increase our knowledge on the patient's perspectives on that matter. The results of this study indicate that a conservative treatment approach is favourable also for TMJ clicking sounds, at least in the short‐term perspective covered in our study.

The treatment modalities tested have two basically opposing approaches. The bite splint has a passivating intention with the aim to decrease parafunctional activity and to alter the biomechanical loading on the TMJ structures. Exercise has an activating approach with the aim to mobilise and challenge dysfunction and pain. Both approaches share the common circumstances that they depend heavily on the patient's compliance, decisions and motivation to complete the treatments in accordance with the instructions and intentions. The supervised exercise model is a method to strengthen the patient's empowerment and self‐efficacy, which hopefully is favourable in a long‐term perspective. Those who were assigned to supervised exercise showed significant improvements also in other indicators of health and well‐being, such as neck disability, mood disturbance and somatisation. These results are in accordance with a previous study on TMD pain patients.^10^ In the study by Yoda et al,^5^ those who reported improvement in their TMJ clicking sounds also reported less discomfort and interference with daily life. The positive effect may thus be assigned both the supportive arrangement and to the exercise in itself. The direct underlying mechanisms are not disclosed by this study but may be related to both unspecified effects of an intervention and changed biomechanical loadings of the structures involved TMJ movements.

The strength of the current study was that the participants were symptomatically reasonably homogenised, randomised to treatment and evaluated intra‐individually by the same examiner who was also blinded to treatment. A weakness was the unbalanced dropout. On the other hand, the higher dropout in the home exercise group may disclose that this choice of intervention calls for a high motivational effort. Our results are thus in line with a previous study on patients with jaw muscle pain that concluded that extensive communication between patient and doctor may be more effective than an occlusal appliance.^11^ Our results are also in accordance with an observational study with counselling and physical therapy that resulted in significant improvement in pain and jaw function in patients with myofascial pain.^12^

## 5 CONCLUSION

Jaw exercise programmes and bite splint therapy had similar positive effects on perceived severity of TMJ clicking sounds. The supervised exercise programme had additional effects on subject's well‐being and may thus help to encourage subjects' empowerment and coping strategies.

## CONFLICT OF INTEREST

No conflict of interest.
